# Immune-Mediated SARS-CoV-2-Induced Orchitis Leading to Complete Testicular Failure: A Report of a Rare Case

**DOI:** 10.7759/cureus.103527

**Published:** 2026-02-13

**Authors:** Michail Stratis, Vasileios Malasidis, Nasos Ioannidis, Menelaos Ioannidis, Ioannis Moysidis, Eleftherios Anagnostou, Theodosia Zegkiniadou, Georgios Chytas

**Affiliations:** 1 Urology, General Hospital of Chalkidiki, Polygyros, GRC; 2 School of Medicine, Aristotle University of Thessaloniki, Thessaloniki, GRC; 3 Urology/Andrology, St. Luke’s Hospital, Thessaloniki, GRC; 4 Urology/Andrology, Private Practice, Thessaloniki, GRC; 5 Urology, Private Practice, Thessaloniki, GRC; 6 Pathology, Istoiatriki, Thessaloniki, GRC; 7 Oral Medicine/Pathology, Aristotle University of Thessaloniki, Thessaloniki, GRC; 8 Male Fertility/Andrology, Zeginiadou Fertility and Andrology Laboratory, Thessaloniki, GRC; 9 Urology, St. Luke's Hospital, Thessaloniki, GRC

**Keywords:** autoimmune orchitis, azoospermia, case report, immunological trigger, sars-cov-2

## Abstract

Autoimmune orchitis is a rare cause of male infertility characterized by immune-mediated impairment of spermatogenesis. SARS-CoV-2 infection has been implicated in testicular inflammation and immune dysregulation, but reports of virus-associated autoimmune orchitis remain exceedingly uncommon. We describe a 40-year-old man with rapidly progressive infertility leading to azoospermia and testicular failure, occurring in the context of a balanced chromosomal translocation that may have conferred genetic susceptibility. Histological findings were consistent with autoimmune orchitis, and serological evidence indicated prior asymptomatic exposure to SARS-CoV-2 in the absence of recent vaccination, suggesting a potential immunological trigger. This case supports the hypothesis of a “second-hit” mechanism whereby virus-induced immune activation may accelerate testicular damage in genetically vulnerable individuals. To our knowledge, only a single-digit number of cases of SARS-CoV-2-associated autoimmune orchitis have been reported. Early fertility preservation, genetic and immunological evaluation, and long-term endocrine follow-up should be considered in men presenting with unexplained, rapidly progressive infertility. Further studies are needed to clarify the relationship between SARS-CoV-2, testicular autoimmunity, and male reproductive health.

## Introduction

Autoimmune orchitis represents a rare but significant cause of male infertility, characterized by immune-mediated destruction of germ cells and progressive spermatogenic failure. The pathophysiology involves dysregulated T-lymphocyte activity, with CD3+, CD4+, and CD8+ T cells playing a central role in mediating testicular inflammation and disruption of immune privilege within the seminiferous tubules [1-3]. Such immune activation can result in the breakdown of the blood-testis barrier, leading to impaired spermatogenesis and irreversible testicular damage.

Since the emergence of SARS-CoV-2, increasing evidence has suggested that the virus may adversely affect male reproductive health through both direct and indirect mechanisms. SARS-CoV-2 has been detected in gonadal tissue, and infection has been associated with impaired spermatogenesis, hormonal dysregulation, and orchitis in some cases [4-6].

Genetic susceptibility may further modulate the risk and severity of testicular dysfunction. Balanced chromosomal translocations are identified in a subset of infertile men and are known to disrupt spermatogenesis, meiotic processes, and genomic stability, often leading to oligozoospermia or azoospermia [7]. In such genetically predisposed individuals, viral or autoimmune triggers can accelerate spermatogenic decline through a “second-hit” mechanism. Autoimmune orchitis has been reported as a post-viral complication in several studies [8-10], but rarely in the context of this mechanism.

The testes express high levels of angiotensin-converting enzyme 2 (ACE2) and TMPRSS2 receptors, facilitating potential viral entry into Sertoli, Leydig, and germ cells [11]. Additionally, systemic immune activation and cytokine release, particularly interleukin-6 (IL-6), can contribute to local inflammatory damage and immune-mediated injury, further exacerbating testicular dysfunction [2,3,11,12].

Although autoimmune orchitis following viral infections has been described in the literature [8-10], cases specifically linked to SARS-CoV-2 remain exceedingly rare. Only a few reports have documented immune-mediated testicular inflammation following COVID-19 infection [4-6], and none, to our knowledge, in individuals with a concomitant chromosomal abnormality. This case highlights the potential interaction between genetic vulnerability and SARS-CoV-2-induced immune activation in the pathogenesis of complete testicular failure, presenting a unique case as far as the known literature is concerned.

## Case presentation

**Figure 1:**
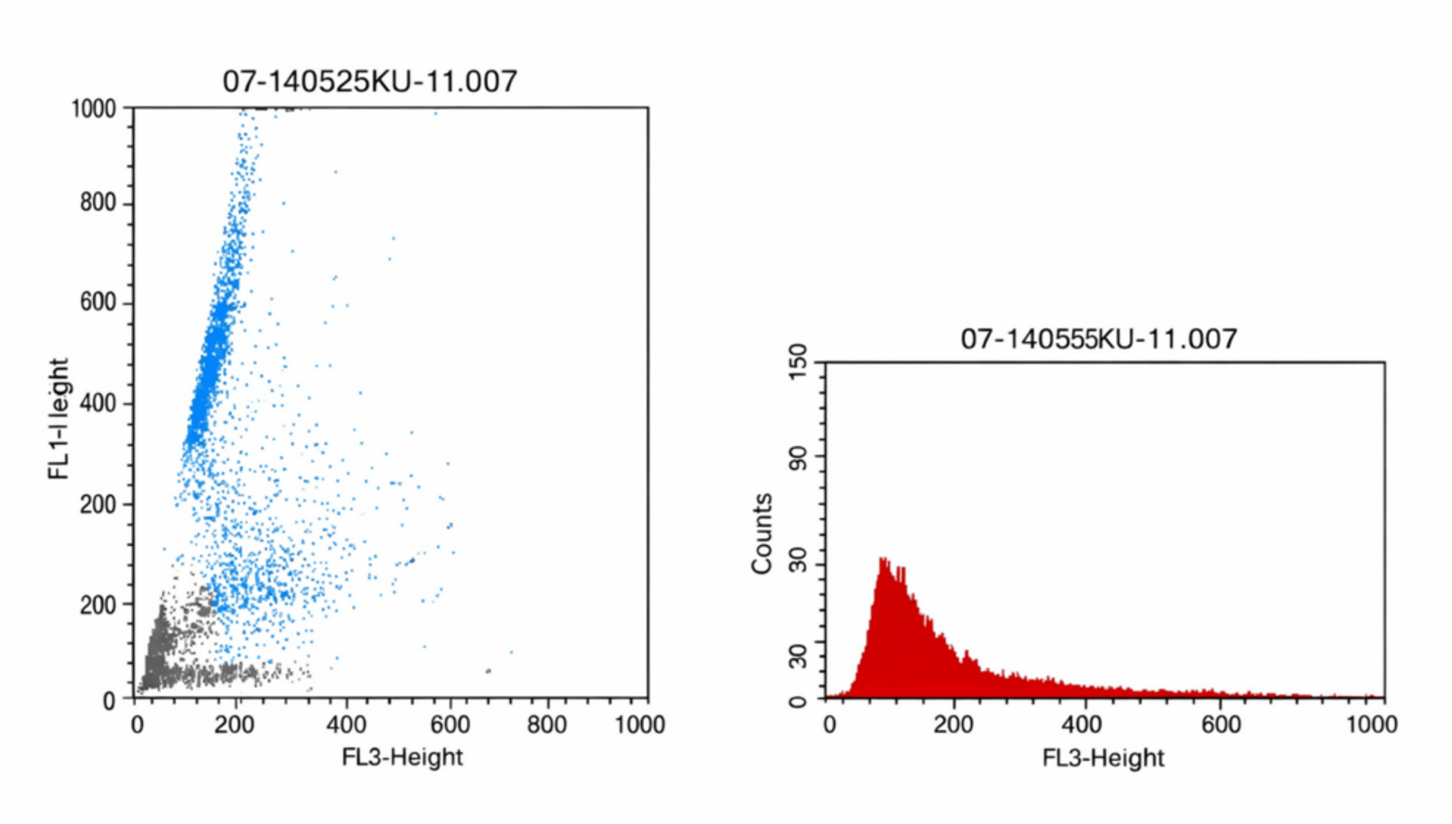
Sperm DNA fragmentation

In July of 2025, a 40-year-old male patient with no family history presented for consultation, reporting infertility issues for the last six months. He had a bilateral varicocelectomy 10 years ago, with no recurrence. The patient reported no history of smoking or exposure to toxins or radiation. The patient had already undergone a sperm DNA fragmentation and three baseline semen analyses: the first one in January of the same year showed slightly decreased sperm mobility and borderline sperm count, while the second and the third one took place one month apart, five months after the initial semen analysis. Those two showed terato- and asthenospermia with progressive oligospermia and a rapid decline of sperm parameters. The sperm DNA fragmentation showed a DNA fragmentation index (DFI) equal to 37%, indicating low DNA quality (Figure 1).

Initially, the patient underwent a testicular triplex and a bilateral ultrasound of the testes, showing a few calcifications of the right testis. At this point, the patient was referred to a fertility clinic for sperm freezing, and at the time, only 440,000 spermatozoa were retrieved. The semen analyses are presented in Table 1.

**Table 1:** Semen analyses of the patient through time

Semen analyses	Date of reference					Reference limit
Variable	January	May	June	July (cryopreservation)	September	N/A
Volume (mL)	2.6	2.3	2.7	2.2	2.1	≥1.5
pH	8.4	8.4	8.4	8.4	8.5	≥7.2
Sperm concentration (x10^6^/mL)	12	3	1	0.2	1 spermatozoon overall	≥15.00
Total sperm count (millions)	31.2	6.9	2.7	0.44	1 spermatozoon overall	≥39.00
Sperm viability (%)	65	64	71	N/A	N/A	≥58
Sperm motility (37°C) – 1h						
Rapid progressive motility (%)	12	0	9	2	0	≥32
Sluggish progressive motility (%)	27	12	19	14	0	N/A
Non-progressive motility (%)	12	17	18	N/A	0	N/A
Immotile spermatozoa (%)	49	71	54	N/A	1	N/A
Morphology (per 200 spermatozoa)						
Normal forms	1	0	N/A	4	N/A	≥8
Head defects	199	200	N/A	N/A	N/A	N/A
Neck defects	79	76	N/A	N/A	N/A	N/A
Tail defects	61	44	N/A	N/A	N/A	N/A
Cytoplasmic residue	9	12	N/A	N/A	1	N/A

Then, the patient was proceeded to hormonal profiling, showing elevated luteinizing hormone (LH), borderline total testosterone, and follicle-stimulating hormone (FSH) (Table 2).

**Table 2:** Hormonal profile FSH: follicle-stimulating hormone; LH: luteinizing hormone

Hormonal analyses	Date of reference		Reference limit
Variable	July	August	N/A
FSH (mIU/mL)	12.1	15.5	1.5-12.4 mIU/mL
LH (mIU/mL)	9.4	11.2	1.7-8.6 mIU/mL
Total testosterone (mIU/mL)	3.17	4.86	2.49-8.36 mIU/mL
Estradiol – E2 (pg/mL)	11.4	N/A	7.6-42.6 pg/mL
Prolactine (PRL) (ng/mL)	11.7	N/A	4.0-15.2 ng/mL
SHBG (sex hormone–binding globulin) (nmol/L)	31.5	N/A	13.5-71.4 nmol/L

**Figure 2:**
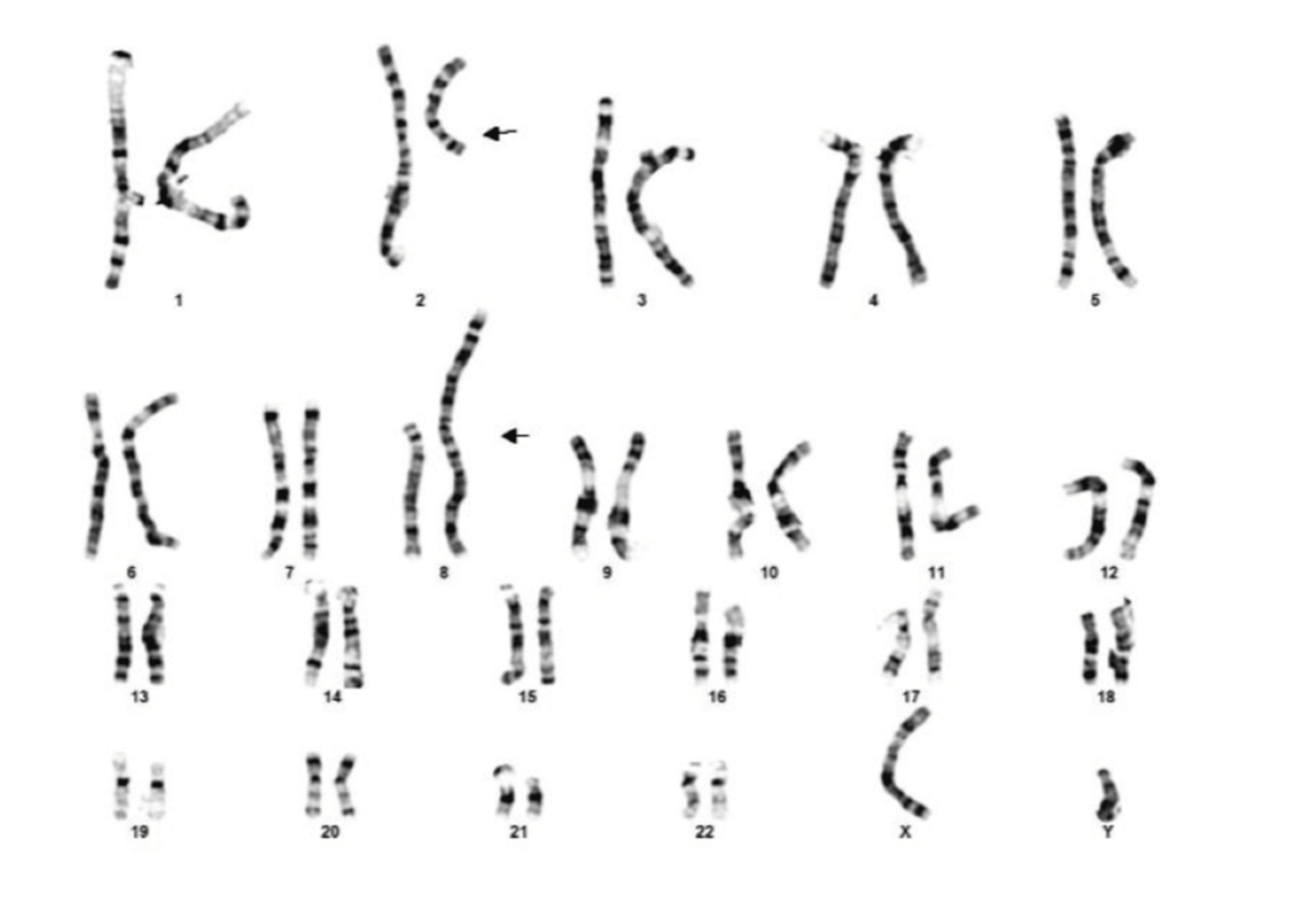
Karyotype, indicating a balanced translocation involving chromosomes 2 and 8

Karyotyping 46XY;t(2;8)(q11.2;p21) revealed a balanced translocation involving chromosomes 2 and 8. The karyotype is shown in Figure 2.

**Figure 3:**
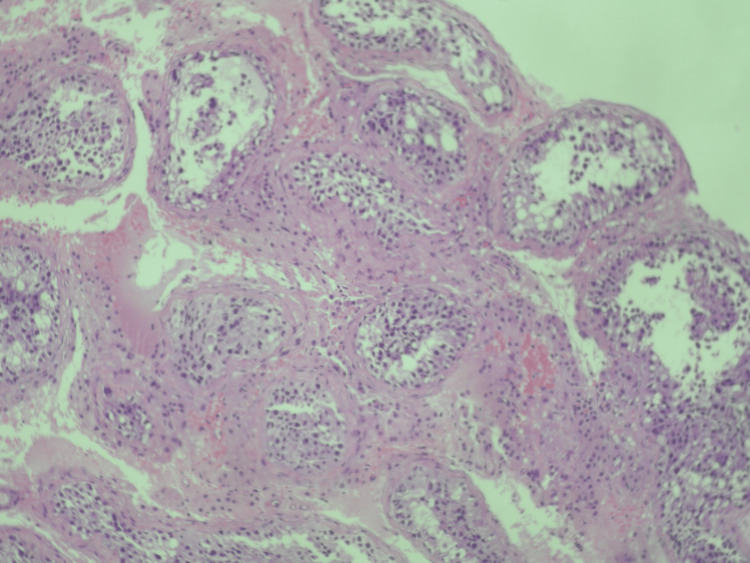
Seminiferous tubules present; hyperplastic Leydig cells noted within the intestitial space (hematoxylin and eosin stain X100) Hematoxylin and eosin staining at ×100 magnification demonstrates preserved seminiferous tubules with prominent hyperplasia of Leydig cells within the interstitial space, consistent with post-inflammatory endocrine remodeling in the setting of SARS-CoV-2–associated immune-mediated testicular injury.

**Figure 4:**
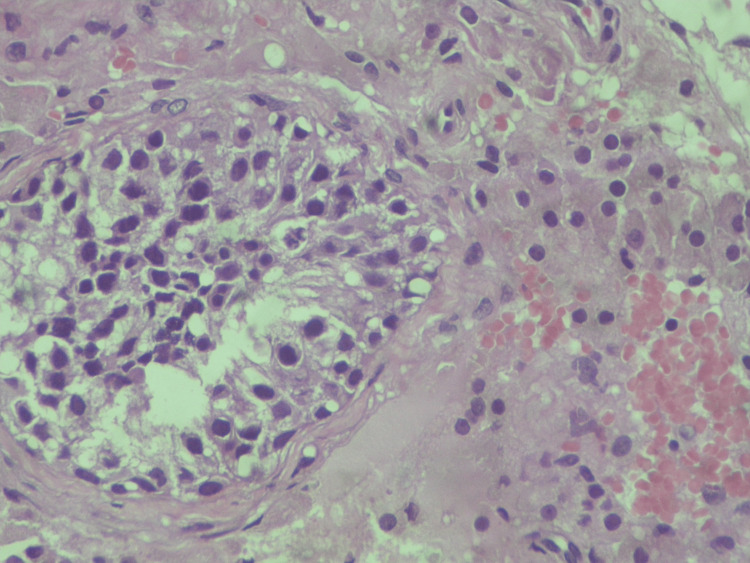
Hypospermatogenesis (hematoxylin and eosin stain X400) Hematoxylin and eosin staining at ×400 magnification demonstrates hypospermatogenesis characterized by reduced germ cell layers and interstitial inflammatory changes, supporting immune-mediated impairment of spermatogenic activity in the context of SARS-CoV-2–associated testicular injury.

**Figure 5:**
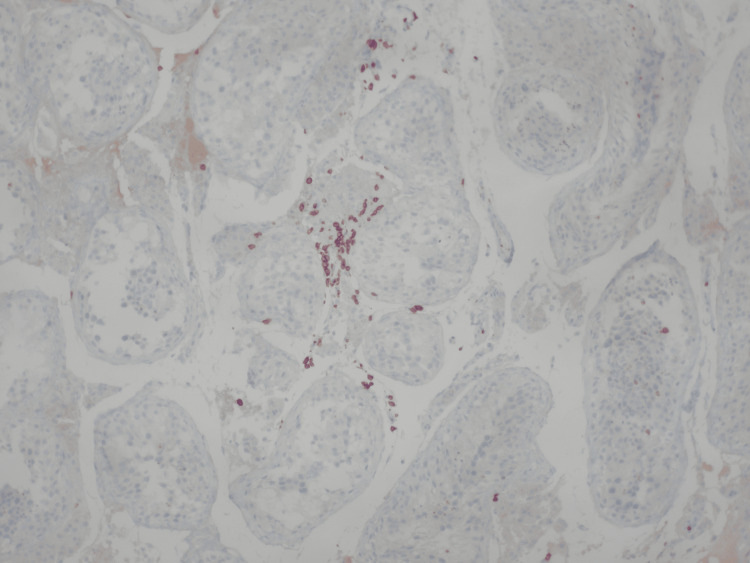
Few scattered and a small aggregate of CD3-positive T-lymphocytes, within the interstitial space (immunoperoxidase X100) Immunoperoxidase staining at ×100 magnification demonstrates scattered and focally aggregated CD3-positive T lymphocytes within the interstitial space, supporting a T-cell–mediated immune response contributing to testicular inflammation and impaired spermatogenesis following SARS-CoV-2 infection.

**Figure 6:**
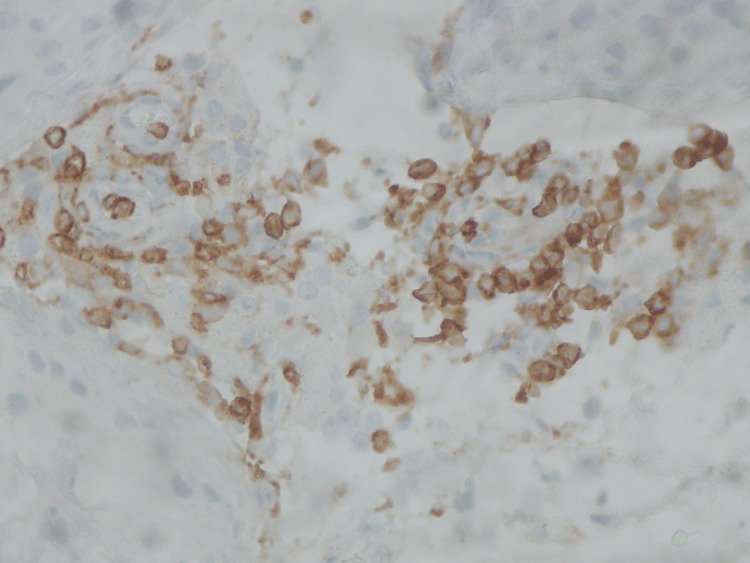
A small aggregate of CD4- positive T-lymphocytes, within the interstitial space (immunoperoxidase X400) Immunoperoxidase staining at ×400 magnification demonstrates a focal aggregate of CD4-positive T lymphocytes within the interstitial space, supporting a helper T-cell–driven immune response contributing to immune-mediated testicular inflammation and spermatogenic dysfunction associated with SARS-CoV-2 infection.

**Figure 7:**
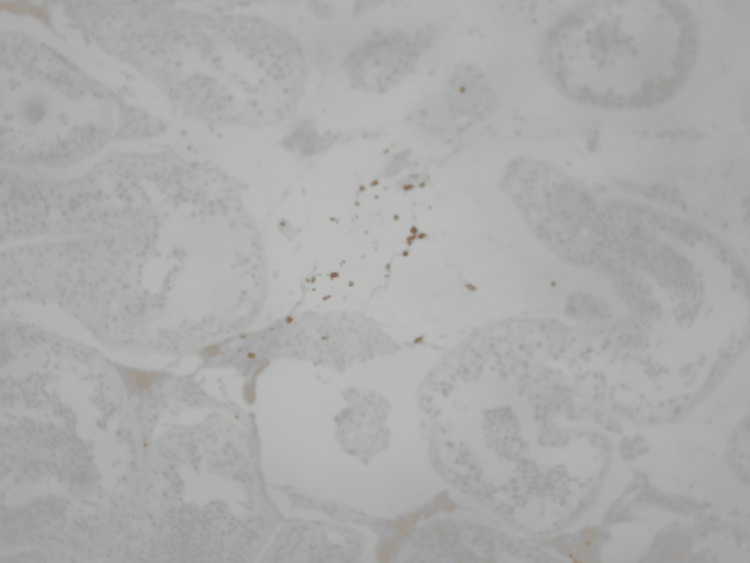
Few scattered and a small aggregate of CD8-positive T-lymphocytes, within the interstitial space (immunoperoxidase X100) Immunoperoxidase staining at ×100 magnification demonstrates scattered and focally aggregated CD8-positive T lymphocytes within the interstitial space, supporting a cytotoxic T-cell–mediated immune response contributing to testicular inflammation and spermatogenic failure in the setting of SARS-CoV-2–associated immune-mediated injury.

We proceeded to testicular extraction (TESE) and biopsy of both testes. Only a few spermatids are observed within the lumen of seminiferous tubules, without the presence of spermatozoa (Figures 3-7).

Due to the increased probability of autoimmune orchitis, the sample was assessed for inflammatory and endocrine biomarkers. The results were positive for CD3+, CD4+, and CD8+ T-lymphocytes within the interstitial space, while no malignancy was detected. These findings were consistent with immune-mediated orchitis, likely subacute, due to the rapid decline of sperm count. Upon research, it was deemed essential to evaluate the patient for underlying systemic autoimmune conditions, which would resonate with the autoimmune reaction and other uropathogens, including viruses known to cause such conditions, such as parvovirus B19, cytomegalovirus (CMV), adenovirus, mumps virus, and SARS-CoV-2. The results of the serological test for SARS-CoV-2 anti-S antibodies demonstrated a high titer of 16841 U/ml, far exceeding the positivity threshold of 0.8 U/ml (Table 3).

**Table 3:** Immunological tests Anti-HBc: hepatitis B core antibody;​ Ab – SARS-CoV-2 -S: antibody to SARS-CoV-2 spike protein;​ Anti-HCV: hepatitis C virus antibody; TPHA: treponema pallidum hemagglutination assay

Examination	Result	Reference limit
Anti-HBc	Negative – 2.15 IV	Neg>1; Pos≤1
Ab – SARS-CoV-2 -S	16841.0 U/mL	Neg<0.8; Pos>0.8
Anti-HCV	Negative – 0.078 IV	Neg<1.0; Pos>1.0
Anti-HIV (I and II)	Negative – 0.20 IV	Neg<1.0; Pos>1.0
TPHA	Negative	Neg<1/80

This result is suggestive of recent exposure to SARS-CoV-2, either through recent infection or recent vaccination. No recent vaccination or COVID-19 symptoms were reported. These findings support the hypothesis that SARS-CoV-2 infection served as a potential immunological trigger, initiating T-cell-mediated damage in a testis already vulnerable due to the underlying balanced chromosomal translocation. The presence of CD3+, CD4+, and CD8+ T-cell infiltration in the testicular tissue strengthens this association [1]. Emerging evidence suggests that COVID-19 can induce a dysregulated immune response, including elevated levels of pro-inflammatory cytokines such as interleukin-6 (IL-6), contributing to what is commonly described as a cytokine storm. IL-6 plays a pivotal role in T-cell activation and has been implicated in tissue-specific immune damage, including in the testes. Such a hyperinflammatory state may serve as a secondary hit in genetically predisposed individuals, amplifying immune-mediated injury [2;3]. One month after the TESE procedure, the patient’s hormonal profile was reevaluated: the total testosterone level was stable, LH and FSH were significantly elevated well above normal reference ranges, indicating progressive testicular failure. Another month afterwards, a semen analysis was held in order to reevaluate the patient’s semen quality. Only one (1) spermatozoon was found in the sample. The results were in line with complete testicular failure and are included in Table 1.

Due to the combination of the rapid decline in spermatogenesis, the absence of sperm on TESE, and the lack of any established pharmacological treatment for such cases, IVF using cryopreserved sperm was considered the most appropriate course of action. In the event of a future decline in serum testosterone levels and the onset of symptoms related to hypogonadism, exogenous testosterone replacement therapy was proposed for long-term hormonal support and quality-of-life maintenance.

## Discussion

This case highlights autoimmune orchitis potentially triggered by SARS-CoV-2 infection in a genetically predisposed male carrying a balanced chromosomal translocation (46,XY,t(2;8)(q11.2;p21)) leading to complete testicular failure and azoospermia. The patient experienced a rapid decline in semen quality, progressing from mild oligospermia to complete spermatogenic failure within months. Histopathology demonstrated T-cell-mediated testicular inflammation (CD3+, CD4+, CD8+ infiltration), while serology revealed markedly elevated SARS-CoV-2 antibodies in the absence of recent vaccination or symptomatic infection. Together, these findings suggest that recent SARS-CoV-2 exposure acted as an immunological trigger, precipitating autoimmune orchitis and accelerating testicular failure in a testis already vulnerable due to an underlying chromosomal mutation. Only a few but notable cases of autoimmune orchitis triggered by SARS-CoV-2 infection have been reported globally [4-6].

As far as genetic factors are concerned, it is easily concluded that there was a baseline susceptibility due to the balanced chromosomal translocation. Specifically, such translocations are detected in a notable percentage of infertile men and are associated with impaired spermatogenesis, meiotic arrest, and an increased risk of oligozoospermia or azoospermia [7]. In this case, the patient’s translocation likely represented a baseline vulnerability, contributing to the gradual deterioration of semen quality over time, explaining the infertility issues that the patient initially faced, which were shown in the initial semen analysis (January 29, 2025). The rapid acceleration of spermatogenic decline, however, suggests the influence of an additional pathogenic factor. Histopathological findings confirmed the presence of CD3+, CD4+, and CD8+ T-lymphocyte infiltration within the interstitial tissue, consistent with autoimmune orchitis. This is a rare but recognized cause of male infertility, characterized by T-cell-mediated damage to the seminiferous tubules and impaired spermatogenesis. Immune orchitis has been reported following trauma, surgery, or infection, and can lead to progressive testicular failure when untreated [8-10].

In this case, SARS-CoV-2 played a crucial role as an immunological trigger. The patient demonstrated markedly elevated SARS-CoV-2 antibody titers in the absence of recent vaccination or symptomatic infection, supporting the possibility of recent viral exposure and asymptomatic infection. Emerging evidence indicates that SARS-CoV-2 may impair male fertility through both direct invasion of testicular tissue and indirect mechanisms, such as immune-mediated inflammation and hormonal dysregulation [11]. Direct effects include viral entry into testicular tissue through ACE2 receptors, while indirect mechanisms involve systemic immune dysregulation and cytokine-mediated damage. Interleukin-6 (IL-6), in particular, is a central mediator of T-cell activation and has been implicated in testicular inflammation. Studies indicate that the testes, particularly spermatogonia, Sertoli cells, and Leydig cells, show high levels of ACE2 expression, making them potential targets of SARS-CoV-2. As a result, the virus may impair spermatogenesis and contribute to the development of orchitis in affected men. From a pathophysiological perspective, viral binding to ACE2 receptors within testicular tissue can trigger local inflammation, which may progress to orchiepididymitis and present clinically with testicular pain [12]. We propose that in this case, SARS-CoV-2 infection acted as a secondary immunological trigger (“second hit”), precipitating immune-mediated orchitis and leading to testicular failure in the context of an already genetically vulnerable testis.

When it comes to clinical implications, this case underscores several important clinical considerations. First, in men presenting with rapidly declining semen parameters, early referral for sperm cryopreservation is critical, as further deterioration may render biological parenthood via assisted reproduction impossible. Second, a comprehensive evaluation, including genetic testing and hormonal profiling, is strongly indicated to be performed in all cases of unexplained infertility. Third, clinicians should remain vigilant for possible testicular involvement in patients with recent SARS-CoV-2 exposure, particularly in those with predisposing genetic or structural abnormalities.

In terms of management, given the absence of mature spermatozoa in TESE samples, combined with the rapid deterioration of spermatogenesis and the lack of targeted pharmacological options for immune-mediated orchitis, the available therapeutic strategies are limited. Consequently, assisted reproductive techniques (ART), such as IVF, using the cryopreserved sperm, represented the most viable approach to preserve the patient’s reproductive potential. Furthermore, given the rising gonadotropin levels and borderline testosterone values, long-term endocrine surveillance was deemed essential. Should the patient develop symptomatic hypogonadism or further decline in serum testosterone, testosterone replacement therapy would be indicated to maintain hormonal balance, metabolic health, and overall quality of life.

While concluding, it is important to consider some limitations. While this case report highlights a plausible link between SARS-CoV-2 exposure and immune-mediated orchitis in a genetically predisposed individual, given the nature of a single case report, causality cannot be definitively established. Other biochemical or autoimmune triggers cannot be excluded. Larger cohort studies and mechanistic investigations are needed to clarify the role of SARS-CoV-2 in testicular immune dysregulation and to identify biomarkers predictive of testicular involvement.

## Conclusions

This case illustrates a rare presentation of autoimmune orchitis likely triggered by SARS-CoV-2 infection in a genetically predisposed male carrying a balanced chromosomal translocation. The rapid progression from mild oligospermia to complete spermatogenic failure underscores the importance of early recognition, genetic evaluation, and timely sperm cryopreservation in similar patients. Although definitive causality cannot be proven, this case highlights the need for clinicians to consider SARS-CoV-2 as a potential immunological trigger of testicular damage, particularly in men with underlying genetic or structural vulnerabilities. Further studies are warranted to clarify this association and guide future management strategies.
